# Supplementary figures and images for: MYL9 deficiency is neonatal lethal in mice due to abnormalities in the lung and the muscularis propria of the bladder and intestine
Source: PLoS One. 2022 Jul 8;17(7):e0270820. doi: 10.1371/journal.pone.0270820 (PMC9269942; doi:10.1371/journal.pone.0270820)

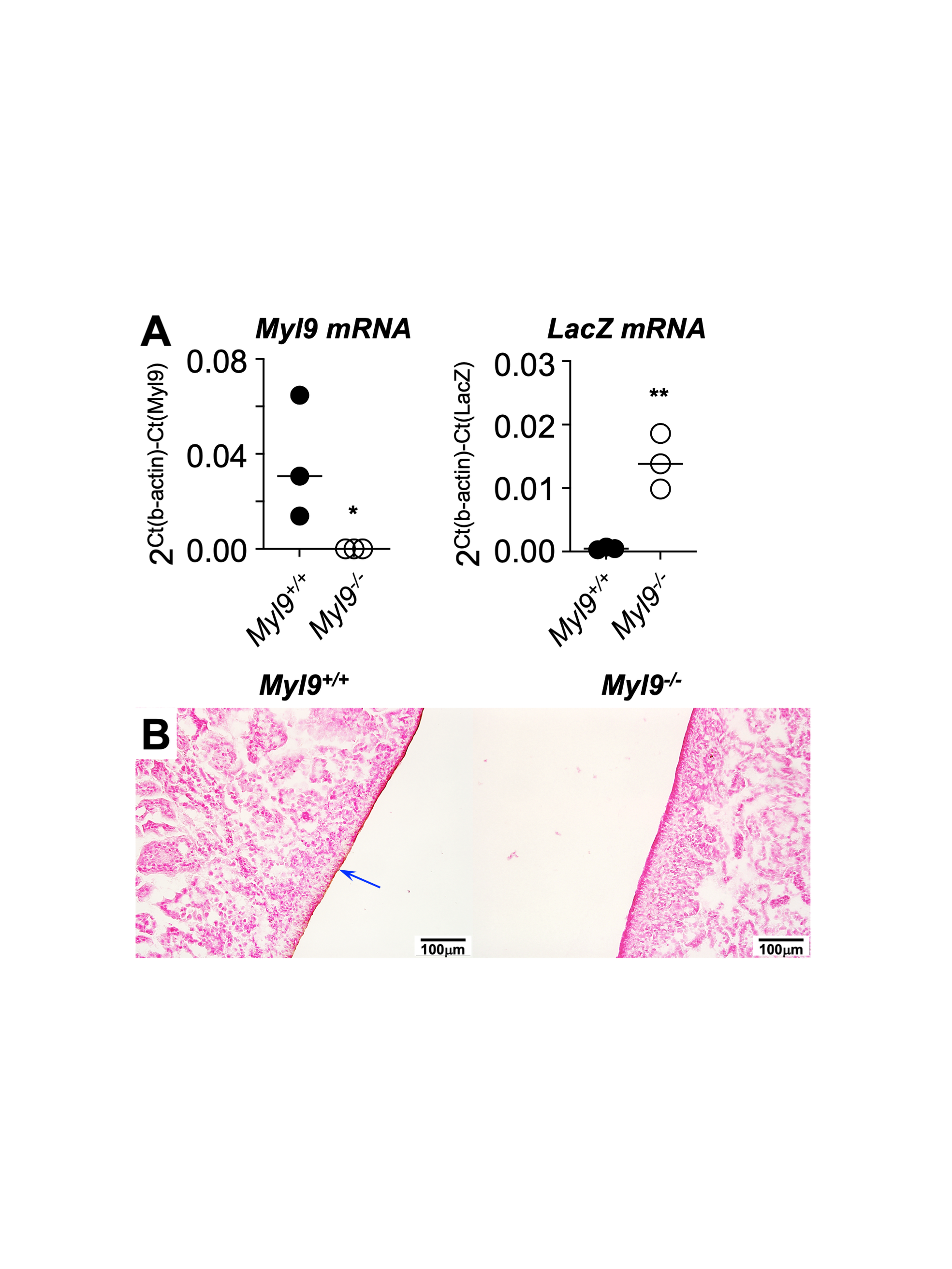

Supplement: S1 Fig — (A) RNA from the small intestine of Myl9+/+ and Myl9-/- pups at D0 post-partum were analyzed for the expression of Myl9 (wildtype) or LacZ mRNA. Each circle is an individual animal, with means indicated by the lines. Comparisons were assessed by t-test (* P < 0.05, **P < 0.005). The individual data points are provided in S3 File. (B) Sections of the jejunum of the small intestine were stained with an anti-MYL9/12A/12B antibody (brown stain) and counterstained with nuclear fast red. The blue arrow indicates staining in the muscularis propria of the Myl9+/+ intestine. (TIF) [file pone.0270820.s001.tif]

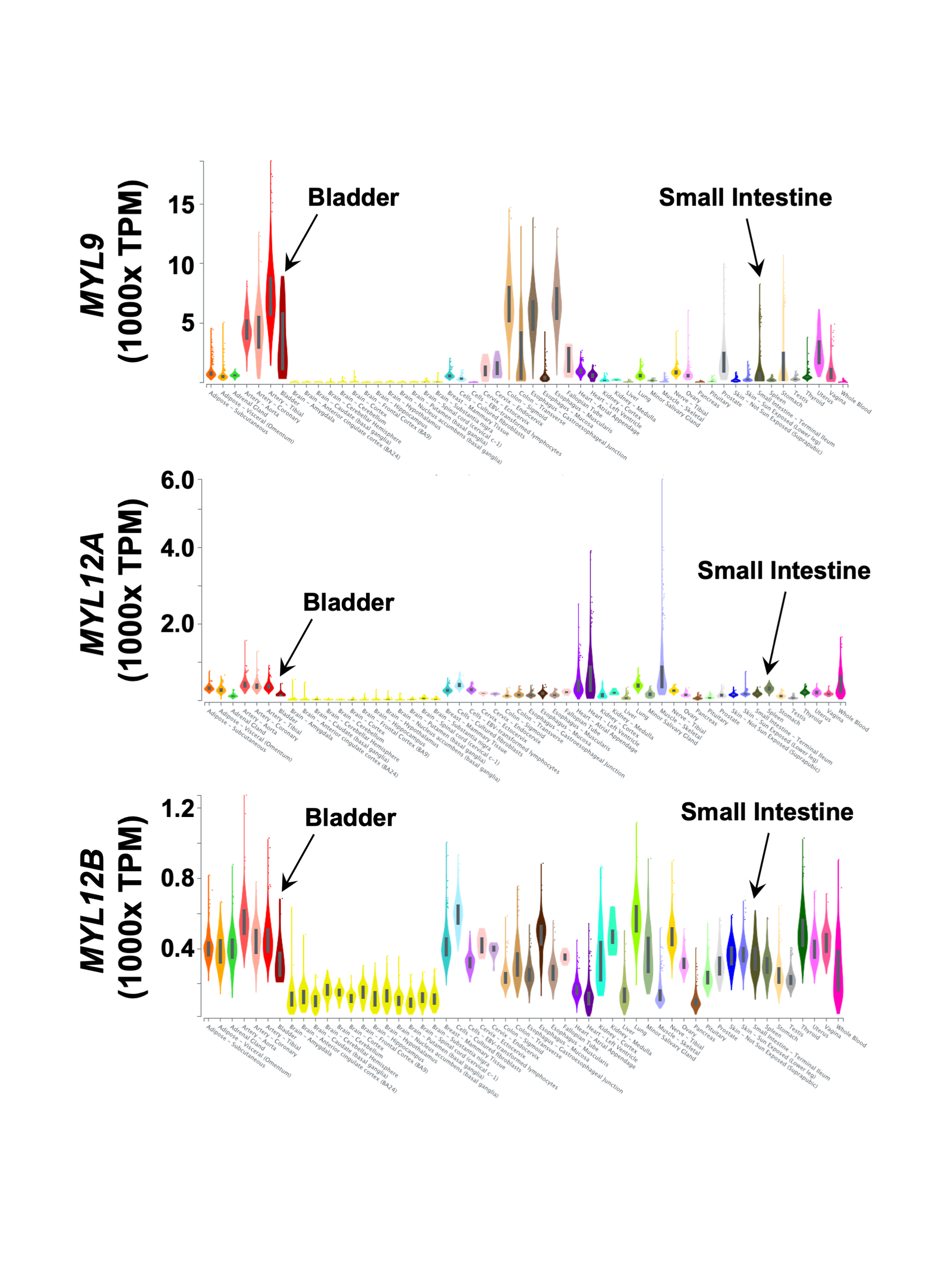

Supplement: S2 Fig — Shown is RNAseq data obtained form the Broad Institute’s GTEx Portal (https://gtexportal.org/home/). Expression is shown as transcripts per million (TPM). (TIF) [file pone.0270820.s002.tif]
