# Supplementary material for: MYL9 deficiency is neonatal lethal in mice due to abnormalities in the lung and the muscularis propria of the bladder and intestine
Source: PLoS One. 2022 Jul 8;17(7):e0270820. doi: 10.1371/journal.pone.0270820 (PMC9269942; doi:10.1371/journal.pone.0270820)
Supplement: Attachment — Submitted filename: Reply to reviewers.docx [file pone.0270820.s006.docx]

A point-by-point reply to editorial and reviewers comments is listed below:

*To comply with PLOS ONE submissions requirements, in your Methods section, please provide additional information regarding the experiments involving animals and ensure you have included details on (1) methods of sacrifice, (2) methods of anesthesia and/or analgesia, and (3) efforts to alleviate suffering.*

The methods for euthanizing mice used in this study have now been included in Methods section under the heading “Mice”.

**Reviewer #1:**

*However, I feel a little confusing about the logic of this paper. The aim of this paper is try to explore the in vivo physiological roles of Myl9 such as hematopoietic stem cells as author mentioned in abstract and introduction. But, the authors did not measure the corresponding functions in MYL9-/- mice. Particularly, the hematopoietic process of the mutant mice was not described or discussed.*

To clarify, the purpose of our study was to understand what the normal physiological function of MYL9 is by studying *Myl9* knockout mice. No where do we state that the purpose of this study is to understand the function of MYL9 in hematopoietic cells. The reference to hematopoietic stem cell failure is simply one of the pieces of background that provides the context for the study. We also refer to cancer and inflammation as examples in regards to the association of Myl9 with various disease processes. We have removed this information from the Abstract in order to emphasize that the purpose of this study is to analyse the phenotype of *Myl9* knockout mice. This background is now only provided in the Introduction section.

*P16 Line 348: the authors claim “from our study, we now know that MYL9 is highly expressed in the smooth muscle of the bladder but not expressed by skeletal muscle”. It is not true, it has been well known that MYL9 is specifically expressed in smooth muscle.*
That paragraph (including line 348) is discussing the issue of antibodies not being able to differentiate between MYL9, MYL12A and MYL12B because of >96% amino acid identity. The study being discussed in that paragraph found that the anti-MYL9/12A/12B antibody pulls down multiple myosin II MHCs, both ones that are found in smooth and skeletal muscle. The fact that MYL9 is specially expressed in smooth muscle means that it is the interactions with smooth muscle MHCs that will be relevant.

*P15: the authors discuss too much about the overlapping phenotypes of MYL9-deficient mice and
other mutant mice as well as gene-mutated patients, because this paper did not provide any data about the interaction of these proteins.*

Discussion of overlapping phenotypes are important because they are directly relevant to understanding the physiologic function of MYL9. Overlapping expression patterns and phenotypes reveal biologic pathways that are involved. We have, however, tightened the discussion a little and removed some less relevant points, such as the mydriasis phenotype of patients.

*Lymphocytes did not express MYL9 as claimed in this report, how about the function in MYL9-deficient lymphocytes.*

We are unclear what the purpose of this suggestion is. If a cell type does not express a gene, how will studying the impact of knocking out that gene reveal any relevant biologic information? Given the mice exhibit a bladder, intestine and lung phenotype, analysing immunologic function would not be logical.

Minor
*1. P3 last line: have role should be have a role.*

*2. P4 line 72: “.. is activity transcribed” means “..actively transcribed”?*

These have been corrected

**Reviewer #2:**

*1. Quantification of knockout*
*There is a severe lack of quantitative information about the knockout of Myl9 in their mice. They mention that genotyping was performed to show the increased LacZ in the place of exons 3 and 4 but do not attach a gel showing that this is indeed the case. This is a minor issue that can be added to supplementary data but should be attached. Even further, the authors could add supplemental sequencing data from the creation of the mouse line ordered from KOMP to increase confidence in successful insertion and removal of active Myl9 expression and genotype.*

To clarify, genotyping was performed with a single set of primers that detects both the wildtype allele and mutant allele. The mutant allele contains an insert that increases the size of the PCR product. This information is in the Methods Section under “Mice”. We have also included additional data to demonstrate loss of MYL9, which is address below.

*Next, considering that the authors' construct removes exons 3 & 4 of the Myl9 gene and replace it with LacZ, this presents an opportunity for quantification. While acknowledging that Myl9 shares extremely high sequence similarity with Myl12a Myl12b, designing primers within/across exons 3 & 4 of Myl9 would be ideal for qPCR quantification of Myl9 mRNA. By choosing primers containing distinct sequences within Myl9 you would likely show a decrease in Myl9 transcripts, even if there is 100% overlap of primers with Myl12a & Myl12b (as there does not appear to be any functional redundancy or compensation). Additionally, by quantifying LacZ mRNA, you could show that the loss of wild-type Myl9 has happened and that LacZ expression has taken it's place.*

We have now included qRT-PCR data that measures the wildtype *Myl9* transcript, using primers that detect exon 4, or the *LacZ* transcript. Data for the bladder has been include in a new figure (Fig 3A). Data for the small intestine has been included in Supplementary Fig 1A. These clearly show that the knockout allele ablates *Myl9* mRNA expression. A description of this results is also included on p10 in the Results section.

*2. Protein Expression of MYL9*
*Fully understanding that Myl9 and Myl12a and Myl12b have incredibly high sequence homology, I agree that detecting Myl9 separate from Myl12a and Myl12b is extremely difficult…*

We have completed immunohistochemical staining for MYL9/12A/12B protein of bladder and small intestine. To our (pleasant) surprise, we were able to detect loss of what we believe is MYL9 protein. Based on the tissue expression databases like the GTEx Project of the Broad Institute (<https://gtexportal.org/home/>), the bladder and small intestine appear to express only low levels of MYL12B and little MYL12A. Thus, the signal detected by the anti-MYL9/12A/12B is likely to be primarily MYL9. Indeed, we found that the antibody signal was largely absent from the tissues of our *Myl9^-/-^* animals. This immunohistochemical data has now been included in Fig 3B and Supplementary Fig 1B, and are also described in the Results section on p10.

*3. Additional FACS*
*The authors use FACS to isolate TCR-beta+ cells to test levels of beta-galactosidase and find a lack of expression, which goes against previous data showing that it is expressed. I feel that this needs to be resolved further with other, more general hematopoietic cell markers such as CD45 or CD34 and then isolating either RNA/protein from these cells and quantifying Myl9/LacZ expression. It is a striking conundrum that the beta-galactosidase is only showing up in smooth muscle but not other known cellular sources. This undercuts their contention that knockout is indeed globally accomplished and is in direct contrast to previous publications. At the very least, further exposition as to why this contrast is occurring is needed.*

The detection in Jurkat cells in the paper by Aoki et al only emploued RT-PCR to show that the transcript is detectable. This was not quantitative. No quantitative approaches, like qPCR or even Western blotting was employed. We too can detect *MYL9* transcript in Jurkat cells. In fact, we originally cloned the human *MYL9* cDNA from these cells (Gu et al, Clin Trans Immunol, 11:e1361). However, expression is at very low levels. Tissue expression databases like GTEx shows highest mRNA expression in arteries, intestine, bladder and heart/aorta, shown in Supp Fig 2 in the revised manuscript).

Most of previous studies that have measured MYL9 expression, such as by RNA, were on whole organs. This includes the data in GTEx portal. As we showed in this study, Myl9 is also expressed by the smooth muscle of major blood vessels. We thus believe it is this MYL9 that is frequently detected in most organs. In the case of protein, as we discussed (and acknowledged by the reviewer) antibodies to against MYL9 will also detect MYL12A and MYL12B. Thus, unless one is sure that the tissue being analysed only expresses *Myl9* RNA, any protein detected may in fact be MYL12A or MYL12B

That being said, we previously showed that *Myl9* transcript is detectable in hematopoietic stem cells and is derepressed in the DROSHA deficient cells (Johanson, Nat Immunol, 16:1134). We have therefore included a broader range of hematopoietic cell types in the FACS-gal analysis (now Fig 7) and discussed these additional data in the final section of the Results. We show that bone marrow LSK cells (containing the hematopoietic stem cells) do indeed exhibit low levels of beta-galactosidase activity as do common myeloid progenitors. However, we do not believe this low level of Myl9 expression is biologically relevant in normal settings. Only upon upregulation due to loss of DROSHA-mediated suppression does Myl9 affect hematopoietic stem cell function, (Johanson, Nat Immunol, 16:1134). i.e. this low level expression is not normally of physiologic importance.

Finally, the fact that the phenotype of these mice entirely phenocopies the Megacystis–microcolon–intestinal hypoperistalsis syndrome suggests that our mice are indeed a true global Myl9 knockout. The phenotypes of both the mice and human patients can be entirely associated to the loss of smooth muscle function.

*4. Poor Image Quality
The cryosection images throughout the manuscript must be of better resolution. By mentioning that hypertrophy is occurring in both the bladder and intestinal muscle layers, the authors have presented another opportunity for quantification that is not present. With clearer images, the authors will be able to show how much hypertrophy is present in both qualitative and quantitative forms. Within the intestinal images, it is unclear what level of hypertrophy is present and without a clearer image and quantification, it will remain unclear.*

Image compression occurred for the initial review. We have included the original high-resolution images.

*5. Minor English Editing
There are a few incorrectly used (abnormal instead of abdominal on pg. 9 of submission) and missing words in the manuscript but this is of minor concern.*

Corrections have been made.
