# Supplementary material for: MYL9 deficiency is neonatal lethal in mice due to abnormalities in the lung and the muscularis propria of the bladder and intestine
Source: PLoS One. 2022 Jul 8;17(7):e0270820. doi: 10.1371/journal.pone.0270820 (PMC9269942; doi:10.1371/journal.pone.0270820)
Supplement: Attachment — Submitted filename: Reply to reviewer.docx [file pone.0270820.s007.docx]

*It has been documented that Myl9 serves as the regulatory light chain (RLC) of smooth muscle myosin and initiates smooth muscle contraction when it is phosphorylated by Mylk. The function of Myl9 is clear at least in smooth muscle. Also, there are reports showing that tissue specific or global deletion of Myl9 causes abolished smooth muscle contraction and hence the dilation phenotypes of hollow organs. The question is what is the new finding of this study?*

Although not stated by the reviewer, we believe that they are referring to the studies from Sun et al (Front Physiol, 11:593966) and Park et al (Biochem J, 434:171). These are two previous studies that are discuss in our manuscript (4^th^ paragraph of introduction and multiple locations in the discussion sections). As we discussed, Park et al.’s study suggests that MYL9 is important for non-muscle myosin. Sun et al’s study does indeed show that the intestine and bladder from smooth muscle-specific (cre/LoxP) MYL9 deficient mice exhibit impaired contraction in vitro. However, these mice had a mild phenotype compared to germline MYL9 deficient mice. Thus, it is unclear whether is due to incomplete conditional *Myl9* gene deletion or if MYL9 is expressed more broadly than just smooth muscle. In fact, neither the Park nor Sun studies provide any evidence that MYL9 is actually restricted to smooth muscle.

As highlighted by the comments of Reviewer 2 (of the initial manuscript), this has been an ongoing issue for the field because of the lack of reagents that can distinguish between MYL9, MYL12B and MYL12B protein. RNA analysis, such as by RNAseq or qPCR, can distinguish between the different transcripts. However, as explained in our response to Reviewer’s 2 comments, previous studies have only been able to measure MYL9 expression in whole organs and have not been able to determine the actual cell types that expresse MYL9. Thus, it has remained unclear whether MYL9 expression is specific to smooth muscle or has a broad distribution, especially since MYL9 is detectable in almost all organs. This includes the data presented in both the Park et al and Sun et al papers referenced above.

Our study definitely shows for the first time that MYL9 expression is restricted to the smooth muscle layer of contracting organs and of bronchi and smooth muscle. Therefore, failure of smooth muscle cell function is likely the cause of the lethality in MYL9 deficient mice and MMIHS patients. To emphasize this point we have modified the abstract to state “…Using this reporter, we show that MYL9 expression is restricted to the muscularis propria of the small intestine and bladder, as well as in the smooth muscle layer of the bronchi in the lung and major bladder vessels in all organs…”

As we acknowledge the last paragraph of the discussion section, this restricted expression may only be in healthy mice as there is evidence that MYL9 is upregulated in a range of disease settings.
